# Clinical characteristics of patients with suspected cardiac chest pain and angiographically normal coronary arteries in a secondary care hospital

**DOI:** 10.1007/s12471-017-0988-x

**Published:** 2017-04-20

**Authors:** T. S. de Lange, R. Y. G Tijssen, P. Damman, P. F. M. M. van Bergen

**Affiliations:** 1 Department of Cardiology, Westfriesgasthuis, Hoorn, The Netherlands; 2 Heart Center, Academic Medical Center, University of Amsterdam, Amsterdam, The Netherlands

**Keywords:** Chest pain, Normal coronary arteries, Troponin

## Abstract

**Background:**

An important number of patients with suspected cardiac chest pain have non-obstructive coronary artery disease. Our purpose was to describe the clinical characteristics of patients with normal or near-normal coronary arteries in routine cardiological practice in a secondary care hospital.

**Methods:**

In 2013, consecutive patients referred for invasive coronary angiography with suspected cardiac chest pain were analysed at a single-centre (Westfriesgasthuis, Hoorn, the Netherlands). Coronary arteries were defined as normal or near-normal if they showed no stenosis or only slight wall irregularities on visual assessment. Patients with a final non-cardiac diagnosis for the chest pain were excluded.

**Results:**

A total of 558 patients were included. Of these, 151 (27%) showed normal or near-normal coronary arteries on visual assessment. This group of patients were significantly more often female (*p* < 0.001), younger (*p* < 0.001) and non-diabetic *(p = 0.002)*. Forty percent of hospitalised patients who had normal or near-normal coronary arteries at coronary angiography showed an elevated troponin.

**Conclusion:**

In routine cardiological practice, around 1 out of 4 patients with suspected cardiac chest pain undergoing invasive angiography had normal or near-normal coronary arteries. We suggest that premenopausal women with suspected cardiac chest pain could be considered for non-invasive coronary imaging as a first step in clinical practice.

## Introduction

Stable angina pectoris and acute coronary syndrome (ACS) are two clinical manifestations of coronary artery disease (CAD). In accordance with clinical practice guidelines, most of these patients are referred for intracoronary evaluation [1–3]. Previous studies show that approximately 10% of non-ST-elevation myocardial infarction (non-STEMI) patients and 30–60% of patients with stable AP have no CAD [4–8]. Moreover, other studies and registries have shown that up to 39% of patients with suspected CAD may have visually normal or near-normal coronary arteries on invasive coronary angiography [9, 10]. More recently, non-invasive imaging techniques have confirmed that about half of patients with a clinical indication for CAD evaluation had no apparent coronary disease [11–13]. However, recent data on normal coronary arteries in invasive angiography in contemporary routine cardiological practice are lacking. In this paper we describe the clinical characteristics of consecutive patients with normal coronary arteries at invasive coronary angiography for suspected cardiac chest pain at a secondary care centre.

## Methods

### Patient population

The study population consisted of patients who were referred for coronary angiography because of suspected cardiac chest pain between January 1, 2013 and January 1, 2014 at the Westfriesgasthuis, a medium large secondary care centre in Hoorn, the Netherlands. Patients with STEMI were excluded because they were directly referred to a primary PCI centre [3]. Peri-procedural patient characteristics, laboratory results, ECGs and data from coronary angiograms were collected retrospectively. This study complies with the Dutch Medical Research Involving Human Subjects Act.

### Data analysis

Based on the results of the coronary angiogram, patients were classified into two groups: 1) patients with visually normal or only minimal coronary artery stenosis, and 2) patients with more extensive coronary disease. The extent and severity of CAD was assessed by routine visual assessment by the operator. Patients were diagnosed with stable angina, unstable angina or non-STEMI based on clinical data [1–3]. One-year outcomes included coronary revascularisation by either PCI or CABG, and survival during follow-up. Outcomes were collected from electronic patient files.

### Statistical methods

Continuous variables were expressed as mean ± standard deviation and categorical data were expressed as numbers (percentage). Differences between patient groups were analysed with a chi-square or a Fisher’s exact test for categorical variables and independent samples t‑test for continuous variables; two-sided probability of less than 0.05 was considered statistically significant. Multivariate analysis was performed using binary logistic regression. Data were analysed using IBM SPSS statistics for Windows version 19.0 (IBM Corp., Armonk NY).

## Results

**Fig. 1:**
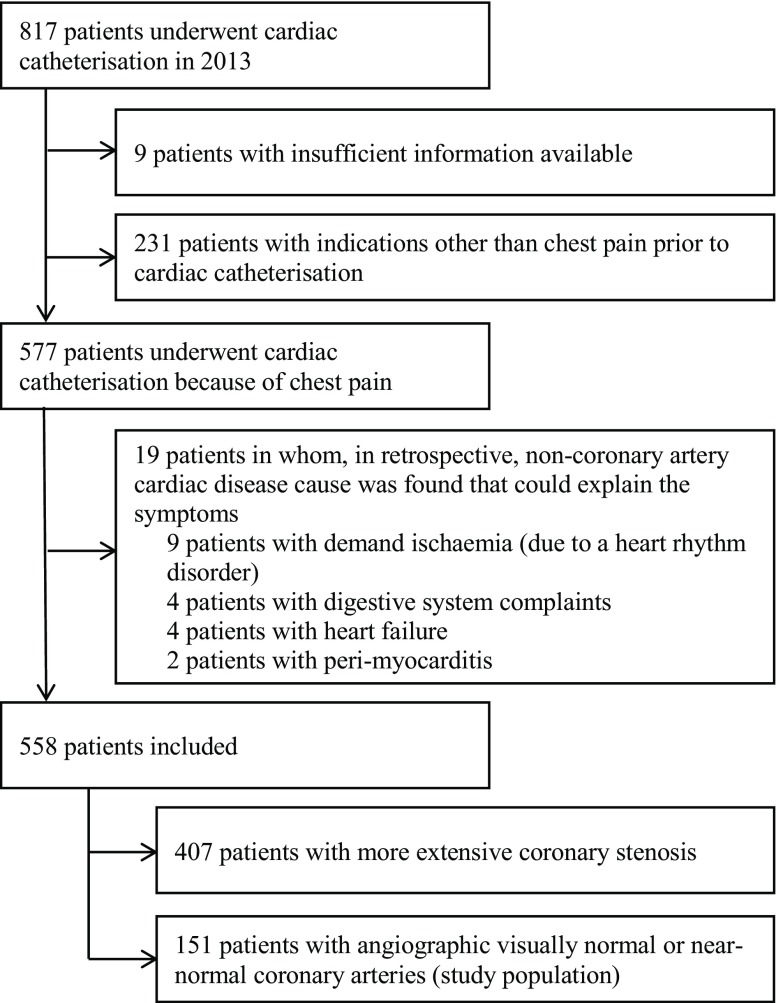
Flowchart

Between January 1, 2013 and January 1, 2014, a total of 817 patients underwent invasive coronary angiography (Fig. 1) of whom 577 (71%) underwent coronary angiography because of suspected cardiac chest pain. During one-year follow-up, 19 patients were excluded because of a definitive non-coronary diagnosis of their chest pain. Consequently, 558 patients were analysed; 151 patients had normal or near-normal coronary arteries, and 407 patients had more extensive CAD (Fig. 1).

Baseline characteristics and 1‑year outcomes of the two patient groups are shown in Table 1. Patients with visually normal arteries showed significant differences compared with patients with more extensive coronary disease with regard to age (62.6 vs 66.7 years, *p* < 0.001), female gender (54% vs 27%, *p* < 0.001) and diabetes (11% vs 23%, *p* = 0.002). Consequent multivariate analysis showed that on coronary angiography lower age, female gender and absence of diabetes were independent predictors of normal coronary arteries.

**Table 1:** Patient characteristics by no angiographic visual abnormalities and 1‑year outcomes

	Group 1		Group 2		*P* Value
	Normal/near-normal coronary arteries (no.)		Coronary artery stenosis (no.)		
Patients – no	151		407		–
Age (y) – mean ± SD	62.6	±11.3	66.7	±9.7	<0.001
Female gender	54	(81/151)	27	(108/407)	<0.001
BMI (kg/m^2^) – mean ± SD	28	±4.5	27	±3.9	0.13
*Risk factors*					
Hypertension	50	(75/151)	53	(214/407)	0.54
Dyslipidaemia	33	(50/151)	38	(153/407)	0.33
Diabetes	11	(17/151)	23	(95/407)	0.002
Positive family history of CAD (<65y)	41	(62/151)	43	(173/407)	0.76
Tobacco use	39	(59/151)	46	(187/407)	0.15
*Diagnostic tests*					
Elevated troponin^a^	40	(36/89)	66	(156/238)	<0.001
Revascularised during follow-up	0.0	(0/150)	30	(118/400)	<0.001
1-year survival rate	99.3	(150/151)	95.8	(389/406)	0.04

*BMI* body mass index, *CAD* coronary artery disease

^a^ Troponin T > 0.013 μg/L with a significant increase in the second value

Table 2 shows the results according to defined clinical diagnosis in both patient groups. All three diagnostic categories encompassed a substantial number of patients with normal or near-normal coronary arteries, predominantly women. None of the patients in the normal coronary artery group had a coronary revascularisation procedure during follow-up versus 30% in the coronary artery stenosis group. Furthermore, one‑year survival rate was 99.3% in the normal coronary artery group and 95.8% in the group of patients with coronary stenosis. The cause of death of the deceased patient in the normal coronary artery group was non-cardiac disease.

**Table 2:** Summary of subgroup analyses according to clinical diagnosis

Subgroup	Group 1 Normal/near-normal coronary arteries		Group 2 Coronary artery stenosis		*P* Value
*Patients with stable angina*					
Patients	27%	(*n* = 62)	73%	(*n* = 169)	–
Female gender	50%	(31/62)	27%	(47/169)	0.002
*Patients with unstable angina pectoris*					
Patients	39%	(*n* = 53)	61%	(*n* = 82)	–
Female gender	51%	(27/53)	23%	(19/82)	0.001
*Patients with non-STEMI*					
Patients	19%	(*n* = 36)	81%	(*n* = 156)	–
Female gender	64%	(23/36)	27%	(42/156)	<0.001

Patients with unstable AP and non-STEMI were all hospitalised

## Discussion

This study shows that approximately 1 out of 4 patients with suspected cardiac chest pain in routine cardiological practice in a secondary care hospital has visually normal coronary arteries on invasive coronary angiography and that these patients are younger, more often female and non-diabetic than patients with more extensive CAD.

### Suspected cardiac chest pain andnormal or near-normal coronary arteries

Several studies have previously reported on patients with suspected cardiac chest pain and normal coronary arteries on invasive coronary angiogram [9, 14–16]. Normal coronary arteries, showing no apparent CAD, are associated with a significantly lower 1‑year risk of myocardial infarction (MI) and all-cause mortality compared with non-obstructive CAD [6]. Our results confirm that the prognosis is better in patients with minimal CAD than in patients with more extensive CAD. More recently, studies using a non-invasive imaging technique such as coronary computed tomography angiography ormyocardial perfusion imaging have confirmed both normal coronary arteries and non-obstructive coronary disease in patients with suspected CAD, with an impaired prognosis in patients with increasing severity of coronary disease [13, 17–22].

Possible causes of chest pain in patients with normal coronary arteries are numerous and include plaque erosion, coronary microvascular disease, endothelial dysfunction, myocardial bridging and coronary artery spasm [23–25]. Forty percent of hospitalised patients with normal coronary arteries in our study population had a clinically significant rise in troponin, indicating myocardial infarction. Earlier studies showed that a prolonged episode of coronary artery spasm can lead to elevated troponin and myocardial infarction [26–28]. Although coronary spasm is sometimes believed to be restricted to Asian patients [29–31], the coronary artery spasm in patients with acute coronary syndrome study (CASPAR) shows that coronary spasm can be a frequent cause of ACS in a European population with an excellent prognosis for survival and coronary events after three years [32, 33].

### Gender differences

Previous studies have shown that chest pain syndromes are more common in women than in men and are less related to the presence of atherosclerosis in the large epicardial coronary arteries [17, 34–37]. Gender-specific factors that affect the development and prognosis of coronary heart disease are diverse but are becoming increasingly clear [38, 39]. Our multivariate analysis confirmed that female gender itself was independently associated with normal coronary arteries in our patients with suspected CAD. Yet, most of the female patients with normal coronary arteries in our patient population were hospitalised for unstable AP or non-STEMI, suggestive of a temporary – but haemodynamically relevant – coronary obstruction.

Vasospasm of the epicardial arteries, microvascular coronary dysfunction (non-endothelial dependent), endothelial dysfunction and higher endothelial shear may all attribute to the higher prevalence of angina and adverse cardiovascular events in women compared with men [40–42]. Our data show that the 1‑year prognosis for patients with suspected CAD and normal coronary arteries is good for both men and women.

### Implications for clinical practice

Recent data show that cardiac MRI or Myocardial Perfusion Imaging (MPI) can help to reduce the use of coronary angiography in a general population of patients with suspected CAD [43]. Our data show that younger premenopausal women with suspected chest pain could be considered for non-invasive coronary imaging as a first step in the diagnostic process.

### Strengths and limitations of this study

This study included consecutive patients undergoing invasive angiography at a non-intervention centre, therefore no additional intracoronary evaluation such as intravascular ultrasound (IVUS) or optical coherence tomography (OCT), could be performed to assess angiographic non-visible atherosclerosis and plaque erosion. Neither could we establish functional coronary disease due to thelack of a spasm provocation test facility at our hospital.

Also, the introduction of highly sensitive troponin assays has improved the accuracy of diagnostic testing for myocardial necrosis and thereby increased the number of patients referred for additional intracoronary examination, in particular in women [44–46]. However, this study reflects a real-life cohort of patients with stable AP or ACS in a secondary care hospital using establised care protocols according to the latest clinical practice guidelines.

### Conclusion

This study demonstrates a high prevalence of normal or near-normal coronary arteries in patients with suspected cardiac chest pain in routine cardiological practice in a secondary care hospital today. These patients were more often female, younger or non-diabetic and had excellent 1‑year survival rate and coronary revascularisation rate

Consequently, we suggest that premenopausal females who have suspected CAD could be considered for non-invasive cardiac imaging as a first step in clinical practice.
